# Towards Understanding Formation Mechanism of Cellular Structures in Laser Powder Bed Fused AlSi10Mg

**DOI:** 10.3390/ma17092121

**Published:** 2024-04-30

**Authors:** Xiaoying Zhang, Xingpeng Zhang, Wenbo Liu, Aoke Jiang, Yu Long

**Affiliations:** 1 School of Resources, Environment and Materials, Guangxi University, Nanning 530004, China; 2115301090@st.gxu.edu.cn (X.Z.);; 2 State Key Laboratory of Featured Metal Materials and Life-Cycle Safety for Composite Structures, Guangxi University, Nanning 530004, China; 3 Institute of Laser Intelligent Manufacturing and Precision Processing, School of Mechanical Engineering, Guangxi University, Nanning 530004, China

**Keywords:** laser powder bed fusion, AlSi10Mg, microstructural evolution, cellular structure

## Abstract

A new approach is proposed that identifies three different zones of the Si-rich network structure (the cellular structure) in laser powder bed fused (LPBF) AlSi10Mg alloy, based on the variation in morphology, grain growth transition, and melt pool solidification conditions. The three identified zones are denoted in the present work as the liquid solidification zone (LSZ), the mushy solidification zone (MSZ), and the heat affected zone (HAZ). The LSZ is the result of liquid–solid transformation, showing small planar growth at the boundary and large cellular growth in the center, while the MSZ is related to a semisolid reaction, and the HAZ arises from a short-time aging process. The boundary between the LSZ and MSZ is identified by the change of grain growth direction and the Si-rich network advancing direction. The boundary between MSZ and HAZ is identified by the start of the breakdown of the Si-rich network. In addition, it is found that the fracture is generated in and propagates along the HAZ during tensile tests.

## 1. Introduction

Additive Manufacturing (AM), a layer-by-layer manufacturing process, overcomes some limitations of conventional methods by allowing complex parts to be formed in one piece, reducing weight while maintaining structural integrity and strength [1,2,3,4,5]. Laser powder bed fusion (LPBF) is one of the metal additive manufacturing technologies that produces metal parts by laser melting metal powders and repeatedly stacking them according to a 3D model [6,7]. Metal powders applied to LPBF include stainless steel [8,9,10], titanium-based alloys [11,12], nickel-based alloys [13,14,15], and aluminum-based alloys [16,17].

Among aluminum-based alloys, AlSi10Mg alloy is a traditional near-eutectic casting alloy with high strength, light weight, good weldability, and excellent corrosion resistance, which is widely used and studied. Conventionally cast Al-Si alloys commonly contain coarse acicular silicon as well as magnesium-containing precipitates, and they do not produce good properties [18]. In contrast, LPBF process can refine the microstructure of AlSi10Mg alloys by rapid solidification (with a cooling rate of 10^3^–10^6^ K/s), thus improving the mechanical properties [19,20,21]. In addition, the microstructure of AlSi10Mg alloys prepared by LPBF is significantly different from that of cast AlSi10Mg alloys. As reported, the cellular structure of AlSi10Mg alloys prepared by LPBF consists of primary α-Al phase islands and a Si-rich eutectic network structure surrounding the former [22,23]. Based on the variation of morphology and size of the network structure, previous studies commonly divided a melt pool into three zones, i.e., the fine-melt-pool (FMP) zone, the coarse-melt-pool (CMP) zone, and the heat-affected zone (HAZ) [24,25,26]. In FMP and CMP, the Si-rich eutectic phase exhibits a network characterized by high interconnectivity, while in HAZ, the network is decomposed and fragmented. There are different reported explanations for the formation of this microstructure. Patakham et al. [27] attributed the changes in the microstructure to the changes in the thermal gradient (*G*) and the growth rate (*R*). Dilip et al. [28] showed that the microstructure of LPBF samples exhibits networks of different sizes as a result of the reheating of the zone adjacent to the melt pool boundary. Yan et al. [29] attributed the difference in the network structure to the different diffusion rates of Si at different sites. Prashanth et al. [30] suggested that the network structure is formed through Bernard—Marangoni-driven instability and particle accumulation structure formation mechanism and that the formation of this microstructure is a result of a combination of high thermal gradient, high grain growth rate, surface instability, and solute transport. Liu et al. [31] showed that the interaction between the laser and the material, and the very high recoil pressure involved tend to cause the propagation of a liquid oscillation or capillary wave, which exacerbates the formation of an inhomogeneous network structure.

Although previous studies have made extensive attempts to rationalize the formation of the microstructure of AlSi10Mg alloys prepared by LPBF, some uncertainties remain unexplained. For example, why does the melt pool boundary show an abrupt change from a coarse cellular structure to a fine cellular structure? Thijs et al. [24] and Li et al. [32] ascribed the microstructural evolution to the solidification condition change from a small *G* × *R* to a large one. However, with this explanation, the transformation of the cellular structure should be a gradual change from coarse to fine, rather than the abrupt change presented by the microstructure of the LPBF AlSi10Mg sample. In addition, the cited studies do not give a clear identification of different zones, and the boundaries of different zones are only delineated in vague ranges based on the morphology and size of the cellular structure. The questions are naturally raised: What are the formation mechanisms of different microstructures presented in LPBF-processed AlSi10Mg alloys? How could we explicitly identify boundaries between different zones? To answer these key questions, this work focuses on careful characterization of the microstructure of LPBF AlSi10Mg alloys, based on which a new approach to identifying three different zones in a melt pool is proposed. They are designated as the liquid solidification zone (LSZ), the mushy solidification zone (MSZ), and the heat affected zone (HAZ). In addition, the formation mechanisms of these zones are also accounted for.

## 2. Materials and Methods

### 2.1. Raw Material Powder and Sample Preparation

**Figure 1:**
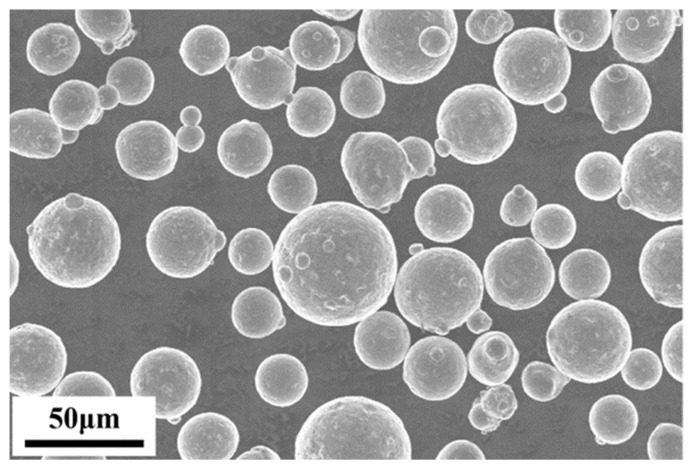
An SEM image of AlSi10Mg feedstock powders.

In this study, AlSi10Mg alloy powder (Avimetal Powder, Beijing, China) prepared by gas atomization was used, the chemical composition of the powder is shown in Table 1, and the particle size ranges from 15 μm to 53 μm. Figure 1 demonstrates scanning electron microscope (SEM) images of AlSi10Mg spherical powder, and the powder shows good sphericity.

**Figure 2:**
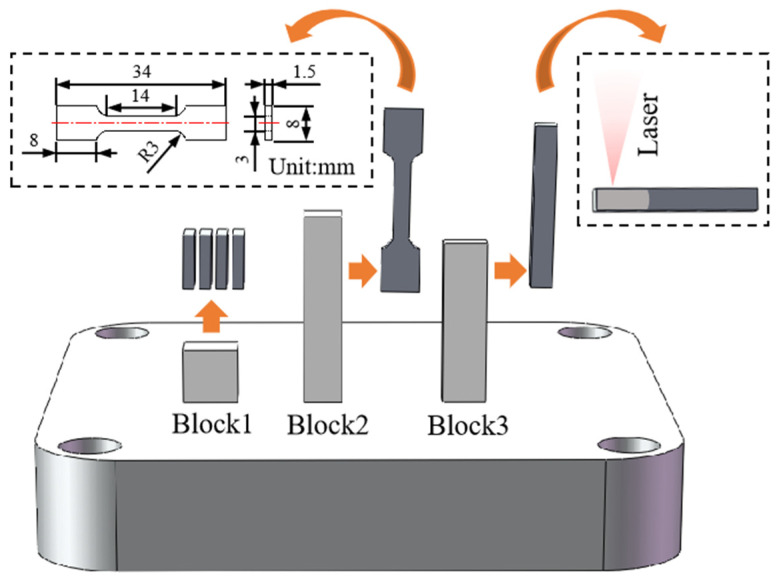
A schematic representation of samples.

LPBF AlSi10Mg samples were made by a Han’s Laser M100 machine (HAN’S LASER, Shenzhen, China) equipped with a 500 W IPG fiber laser and an inert gas protection system. During preparation, high purity argon gas (99.999%) was purged to keep the oxygen concentration in the LPBF chamber below 500 ppm, suppressing unwanted reactions with environmental gases (formation of oxides). After pre-process optimization, the laser beam power was set to 150 W, laser scanning velocity to 400 mm/s, the layer thickness to 50 μm, the hatch spacing to 75 μm, and the rotation direction between consecutive layers to 90°. Three cubic samples were prepared, including block1 (7 × 10 × 10 mm), block2 (7 × 7 × 34 mm) and block3 (8 × 4 × 30 mm), see Figure 2.

In order to verify the trend of morphology change of the Si-rich network affected by heat, we conducted laser remelting experiments. Electrical discharge machining (EDM) wire cutting technique was used to cut Block3 into a 4 × 4 × 30 mm cube. The RFL-C6000X fiber laser was used to irradiate one end of the sample (see Figure 2) with a laser power of 1000 W and a dwell time of 5 s.

### 2.2. Characterization

Block1 was used for optical microscope (OM), scanning electron microscope (SEM), electron backscatter diffraction (EBSD), transmission electron microscope (TEM) and X-ray diffraction (XRD) studies. For OM, SEM and EBSD samples, they were grinded using 150, 500, 1000, 2000 and 3000 grit sandpaper. After grinding, the samples were mechanically polished with 1.0 μm and 0.3 μm alumina suspensions. The samples were then vibrationally polished on a vibratory polishing machine (MEGA INSTRUMENTS UniPOL V0900, Suzhou, China) using successively 0.5 μm diamond suspension and 0.02 μm colloidal silica suspension. After polishing, the surfaces of the samples were etched using Keller’s reagent (2.5 vol% HNO_3_ + 1.5 vol% HCL + 1 vol% HF + 95 vol% H_2_O) at room temperature. For the XRD sample, the same grind and polish as for the OM sample was used, but no etching was used. Block2 was used for tensile testing. The tensile sample was cut from the cube through the EDM wire cutting technique, and the dimensions of the tensile sample are shown in Figure 2. The fracture surfaces and fractures after tensile testing were observed by SEM. The laser remelting samples obtained from Block3 were processed in the same way as the block1 samples and used for SEM observation.

OM analysis was carried out on a metallographic microscope (Motic KPA53MET-BD). SEM analysis was performed on a scanning electron microscope (ZEISS Sigma 300) with an operating voltage of 5 kV. EBSD analysis was performed using ZEISS Sigma 300 equipped with an electron backscatter diffraction detector, at an acceleration voltage of 20 kV, working distance of 20 mm and step size of 0.2 µm. STEM observation and energy dispersive spectroscopy (EDS) analysis were performed at 300 kV using a field emission transmission electron microscope (FEI TECNAI G2 F30). XRD tests were carried out on an X-ray diffractometer (BRUKER D8 DISCOVER) with an operating voltage of 40 kV, an operating current of 40 mA, and a diffraction angle 2*θ* from 25° to 85°. The tensile tests were performed on a WDW electronic universal testing machine, and the sample was stretched at a loading rate of 0.5 mm/min.

## 3. Results

### 3.1. Meso-Structural Characteristics of LPBF AlSi10Mg

**Figure 3:**
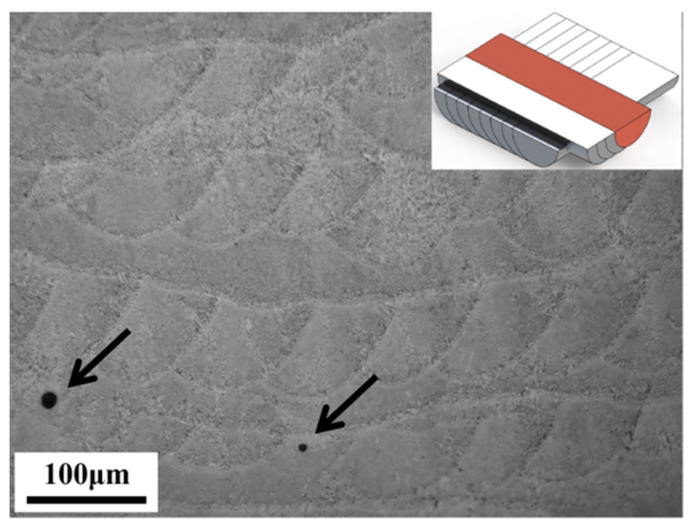
An OM image of the cross-section of LPBF AlSi10Mg alloy.

Figure 3 shows mesoscopic structural features of a cross-section of LPBF AlSi10Mg alloy. A “fish scale” pattern of the melt pools can be seen, which is a direct result of stacking melt pools track by track and layer by layer, as the inset of Figure 3 explains this schematically. In addition, it is revealed that a highly dense, crack-free part is attained, with only a few pores left on the displayed cross-section, marked by the arrows. Considering the small size of ~5 μm, a round shape, and a seemingly random distribution, the pore holes are thus regarded as gas pores, which are inherent to LPBF processes [33].

### 3.2. Microstructural Characteristics of Different Zones

**Figure 4:**
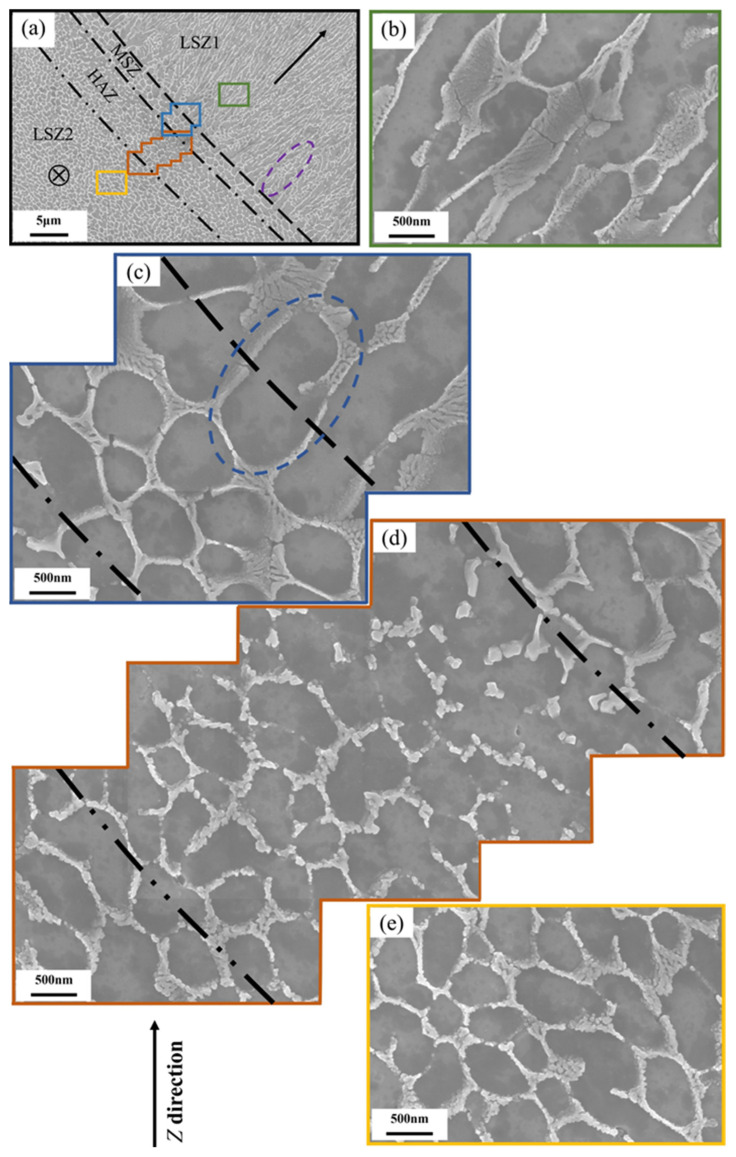
The typical microstructure of the LPBF AlSi10Mg sample. (**a**) An overview of microstructure near a melt pool boundary; in which four different zones can be identified, i.e., (**b**) LSZ1, (**c**) MSZ, (**d**) HAZ, (**e**) LSZ2.

**Figure 5:**
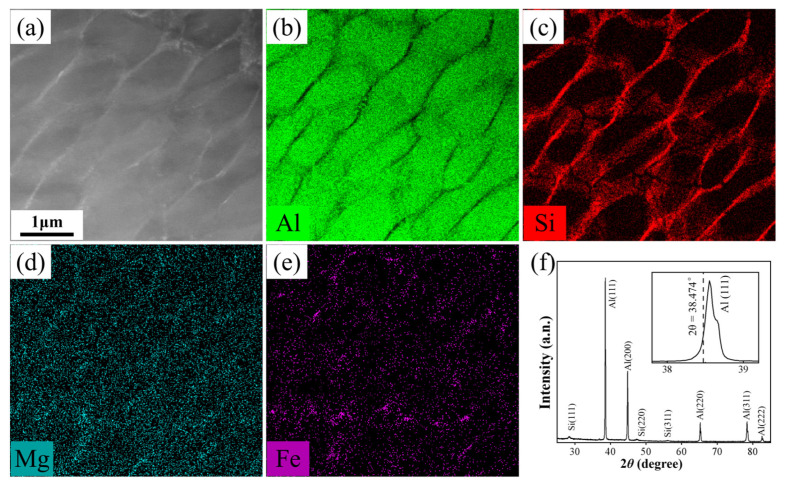
The elemental and phase analysis of LPBF AlSi10Mg sample. (**a**) A STEM image of network structure; STEM-EDS elemental maps of (**b**) Al, (**c**) Si, (**d**) Mg and (**e**) Fe for the same area in panel (**a**); (**f**) XRD spectrum.

Figure 4 exhibits the details of the Si-rich eutectic network near a melt pool boundary of the LPBF AlSi10Mg sample (the Si-rich eutectic network with a brighter contrast and the α-Al matrix with a darker contrast; the elemental analysis will be given in Figure 5). As shown in Figure 4a, the different types of black lines identify different zones (the basis of the identification will be given in Section 4.1): the liquid solidification zone (LSZ), the mushy melt pool solidification zone (MSZ), and the heat affected zone (HAZ). Figure 4b–e present close-ups of them. Since two LSZs exist in the field of view, we designate them as LSZ1 and LSZ2. The probable growth directions are marked in each LSZ, based on the elongation direction of the network structure. Specifically, the elongated network structure in LSZ1 indicates a growth direction running from the center to the upper right corner, perpendicular to the melt pool boundary and towards the interior of the melt pool. On the other side, the more equiaxed network structure in LSZ2 implies a growth direction perpendicular to the plane of paper. It is not completely present in LSZ1 as a regular elongated network structure, and there is also an unstable cellular structure, as shown in the purple dashed area of Figure 4a. The high magnification SEM images (Figure 4b,e) show that the Si-rich network in LSZ1 and LSZ2 exhibit the fibrous-like structure.

Figure 4c shows an enlarged view of MSZ (the blue box in Figure 4a), featuring a larger circular cellular structure than that of LSZ2. Moreover, there exists a larger Si-rich network structure between LSZ1 and MSZ, as shown in the blue dashed area of Figure 4c, and this larger Si-rich network structure can also be seen in previous studies [34]; it is the result of planar growth on the Si-rich network structure of MSZ.

Figure 4d shows an enlarged view of the HAZ (the brown box in Figure 4a). Along the direction of heat transfer (the upper right to the lower left), the Si-rich network structure shows microstructural evolution from large spherical particles to small spherical particles, to a broken spherical Si-rich network, and to an interconnected spherical Si-rich network. It is worth mentioning that despite being broken more or less, the pattern of the Si-rich network is inherited by that of LSZ2. Thus, microstructural evolution can be understood as the effects of a heat treatment. Comparable to previous reports, with the increase in temperature and holding time of a heat treatment, the continuous Si-rich network structure becomes gradually broken [35,36,37].

Figure 5a–e shows a STEM image and the corresponding STEM-EDS elemental maps of the network structure. It is seen that the network structure is rich in Si and the matrix (the cells) is rich in Al, while Mg seems to be uniformly dispersed. The XRD spectrum shown in Figure 5f confirms the presence of FCC α-Al phase and diamond cubic Si phase. Combining STEM and XRD results, it is concluded that the LPBF part consists of FCC α-Al matrix and the Si-rich network structure. Furthermore, it is worth pointing out that the diffraction peaks of FCC α-Al phase shift to the right of standard values. For example, 2*θ* for the present Al (111) peak is 38.555°, higher than the reference value of 38.474° (PDF#85-1327), see the inset. Diffraction peak displacements may be explained by the supersaturation of solute atoms. High cooling rates inherent to LPBF inevitably cause extensive dissolution of the main solute element Si in the α-Al matrix, leading to contraction of the lattice constant, which is eventually reflected by an increased 2*θ* value. This finding is consistent with previous studies [22,38,39,40].

**Figure 6:**
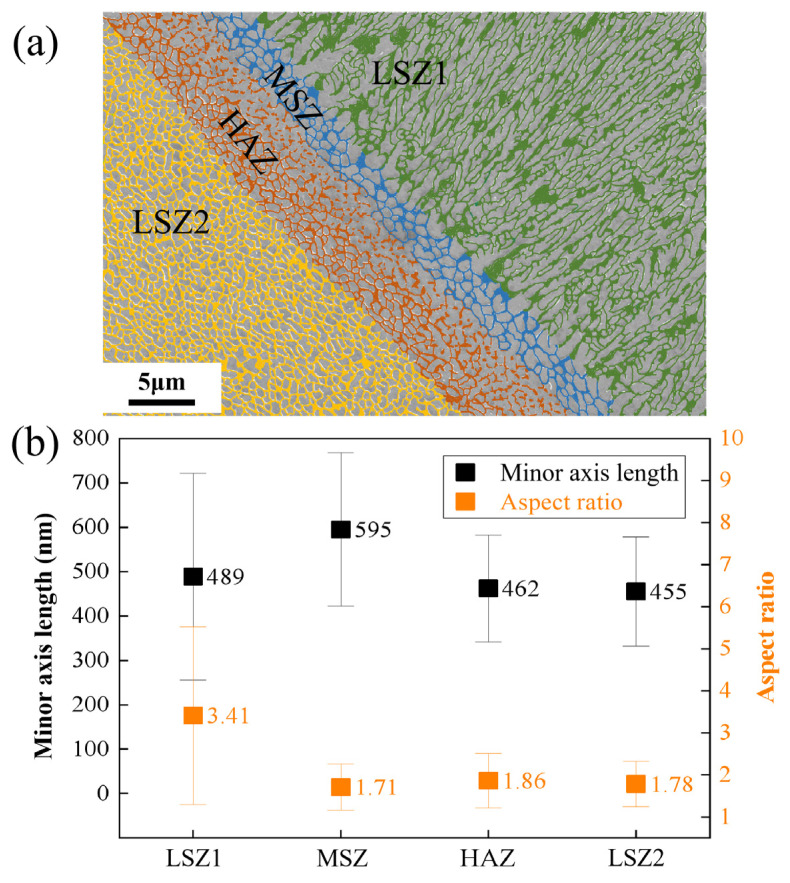
Geometrical characteristics of the Si-rich network structure in different zones in Figure 4a. (**a**) The processed image with the network highlighted; (**b**) the minor axis length and the aspect ratio for the Si-rich network structure in different zones.

The dimensions of cells surrounded by the Si-rich network structure in each zone in Figure 4a were estimated with the help of ImageJ (1.48v) software. Figure 6a shows the processed image and the different colors indicate different zones. Each closed cell is treated as an ellipse, where the Si-rich network in HAZ is partially broken and does not have a defined network, so its size statistics are for reference only. Figure 6b shows the average minor axis lengths of cells in different zones. The average minor axis lengths of LSZ1, HAZ and LSZ2 are comparable, being 489 nm, 462 nm and 455 nm, respectively, whereas MSZ has a larger average minor axis length (595 nm). Figure 6b also shows the aspect ratio of the major axis length/the minor axis length. MSZ, HAZ and LSZ2 have comparable average aspect ratios, being 1.71, 1.86, and 1.78, respectively. The comparable minor axis lengths and aspect ratios of the cell in HAZ and LSZ2, can be explained by the fact that the HAZ was originally part of LMP2 that was subsequently affected thermally when a new neighboring track containing LMP1 was formed. In comparison to LSZ2, MSZ has a larger minor axis length and a comparable aspect ratio. It can be inferred that the Si-rich network in MSZ, which was also part of LSZ2, experienced coarsening during the formation of the neighboring track. The aspect ratio of LSZ1 reaches a maximum of 3.41, which is a result of the elongated network structure aligned with the local growth direction.

**Figure 7:**
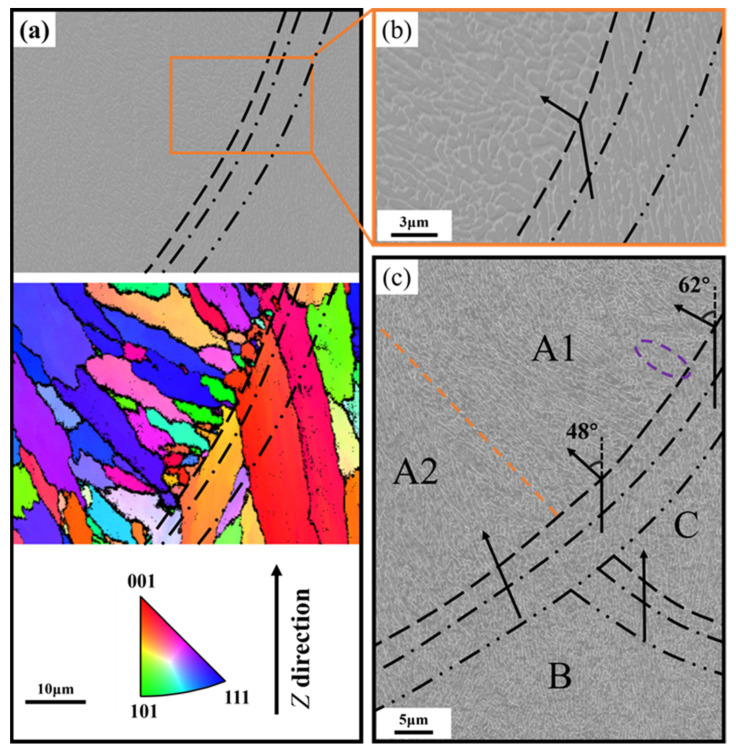
The transition in grain growth direction and the change in network structure occurring at the melt pool boundary. (**a**) An SEM image of melt pool boundary area and the corresponding EBSD IPF map; (**b**) a close-up of the orange box in panel (**a**); (**c**) the network structure in a tri-junction region.

Figure 7 shows the SEM and EBSD results near a melt pool boundary area. The different zones are divided by different types of black lines (the division is the same as in Figure 4a), and the positions of the black lines are the same on the different pictures in Figure 7a and Figure 7b. The EBSD shown in Figure 7a confirms the presence of larger columnar grains and finer grains, and these finer grains are arranged along the melt pool boundary, which is marked with a black dashed line. The high magnification SEM images (Figure 7b) show that the melt pool boundary crosses an array of large cells, as previously highlighted by the blue dashed circle in Figure 4c, and this boundary corresponds to the change of advancing direction of cellular structure. The elongated network structure to the right of the melt pool boundary indicates an upward growth direction, while the left growth direction running towards the upper left.

Figure 7c shows an SEM image of the intersection of three melt pools (A, B, and C). The extension of the boundary of melt pool C (the orange dashed line) divides melt pool A into two parts, denoted by A1 and A2. The growth directions of melt pools A, B, and C are represented by the respective black arrows. When the local heat transfer directions in two adjacent melt pools are similar, the transition of network structure development across the melt pool boundary seems seamless, see, e.g., between A2 and B, or between B and C. In contrast, when the local heat transfer directions in two adjacent melt pools are distinctly different, the transition may require an abrupt change in the advancing direction of the network structure, see, e.g., between A1 and C. In addition to this large-angle change (48° to 62° in the case), the abrupt transition is also accompanied by a period of unstable growth, see the purple dashed circle. Analogous unstable growth was marked by the purple dashed circle in Figure 4a. Roughly, the scale of unstable growth becomes longer with an increased angle of growth direction change.

### 3.3. The Microstructural Evolution of the Si-Rich Network in HAZ

In order to explore how the Si-rich network structure evolves in HAZ, we carried out a laser remelting experiment on an LPBF sample, using a laser power of 1000 W and a dwell time of 5 s, see Section 2.1. The high laser power and the relatively long dwell time used here should have caused a much lower thermal gradient near the melt pool boundary in relation to that in an LPBF process. Thus, the HAZ in the laser remelted sample was significantly expanded, quite advantageous for presentation of microstructural evolution.

**Figure 8:**
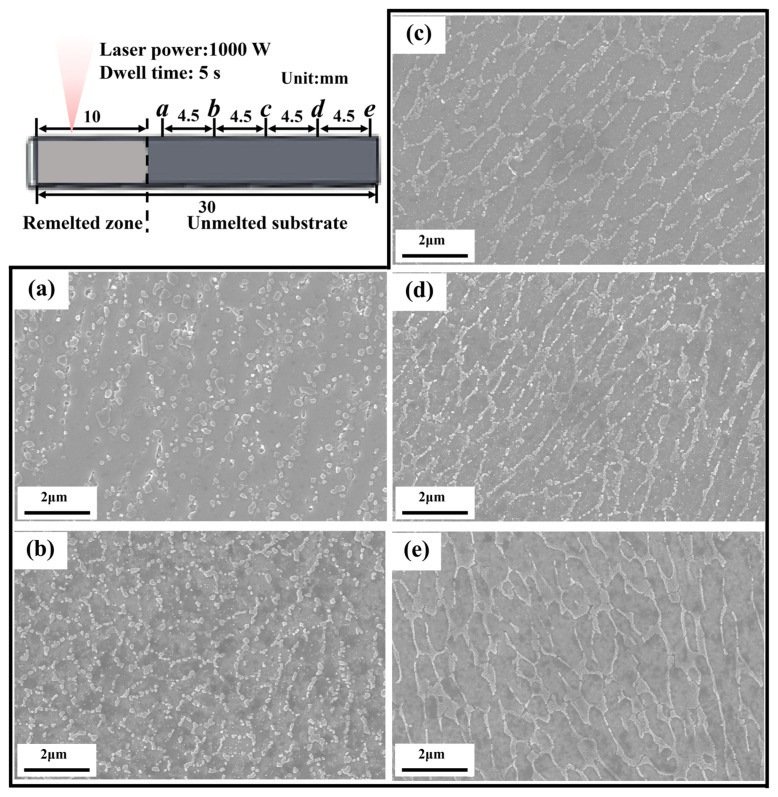
Microstructural evolution in the laser remelted LPBF AlSi10Mg sample with the distance from the remelted zone. (**a**–**e**) SEM images of the evolved microstructure at positions ***a***–***e***, respectively.

Figure 8a–e shows the evolution in microstructures in the unmelted substrate progressively farther away from the remelted zone. It is seen that at a place far enough from the remelted zone, see position ***e***, an intact network structure is retained. Approaching the remelted zone, the network structure becomes gradually broken (***d* → *c* → *b***), and finally the interconnected structure turns to separate round particles, see position ***a***. The microstructural evolution of the Si-rich network structure is consistent with that present in the narrow HAZ in LPBF samples, see, e.g., Figure 4d. In addition, the microstructural evolution is highly analogous to what occurred to aged LPBF AlSi10Mg alloys, see, e.g., the references [41,42]. This is understandable, since all of them have the same root in thermal effects.

### 3.4. Fracture Surface Morphology

**Figure 9:**
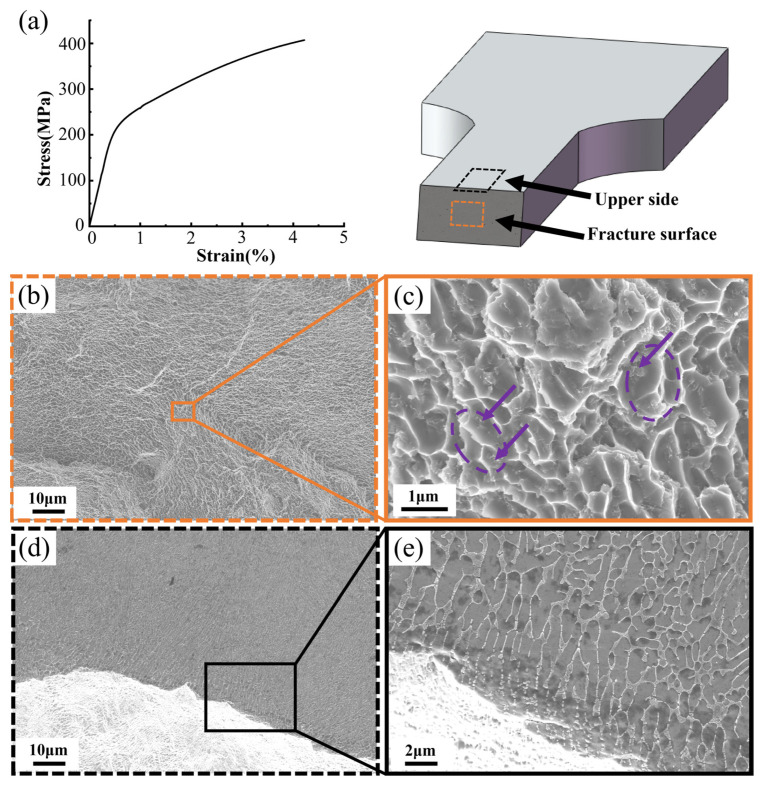
Fracture surfaces of a tensile test sample. (**a**) The stress–strain curve; (**b**,**c**) SEM images of the fracture surface (circled by the orange dashed line); and (**d**,**e**) upper side near the fracture surface of the tensile sample (circled by the black dashed line).

A tensile test was carried out in order to investigate the weak zone in the LPBF AlSi10Mg sample. Figure 9a shows the stress–strain curve of a test sample cut parallel to the building direction, with a tensile strength of 407 MPa and a breaking elongation of 4.3%. The fracture surface shows a fracture pattern of flat steps with less deformation (Figure 9b). And the Si-rich phases can be found on fracture surface under a higher magnification (Figure 9c), which is consistent with a previous finding [43]. The microstructure of the sample’s upper side near the fracture surface is shown in Figure 9d,e. Clearly, an area containing broken spherical Si particles is disrupted by the fracture (Figure 9e), suggesting that the fracture occurred in the HAZ.

## 4. Discussion

### 4.1. Identification of Different Zones

According to the previous discussion, the microstructure of the melt pool can be divided into the LSZ, MSZ, and HAZ. Compared to the ill-defined delineation of the different zones of the melt pool in previous studies, this study identifies the three zones more precisely through detailed observation and analysis. Among them, LSZ and MSZ are separated by grain growth and change of advancing direction (Figure 7a), which also corresponds to the change of advancing direction of cellular structure (Figure 7b). It can be clearly found that the boundary of LSZ and MSZ correspond to the initiation between the new orientation of the finer grains, and the primary α-Al phases nucleate at the fusion line and grows into the melt [27]. There is a partial remelting of the matrix meta with no nucleation barrier, and epitaxial growth occurs when the grain orientation of the base coincides with the direction of the heat transfer, otherwise new nuclei will be formed [44]. Only a few grains can grow further towards the interior of the melt pool, where small newly nucleated grains are also the product of competitive growth [45]. MSZ and HAZ can be identified by the broken Si-rich network (Figure 4d). No clear identification can be given for the boundary of HAZ, as it was subject to a minimal heat effect. The heat conduction of the melt pool can be transferred to multiple other melt pools, leading to the structural evolution of the Si network. The boundary of HAZ is set according to the evolution law of the Si-rich network.

### 4.2. Microstructural Formation of Different Zones

**Figure 10:**
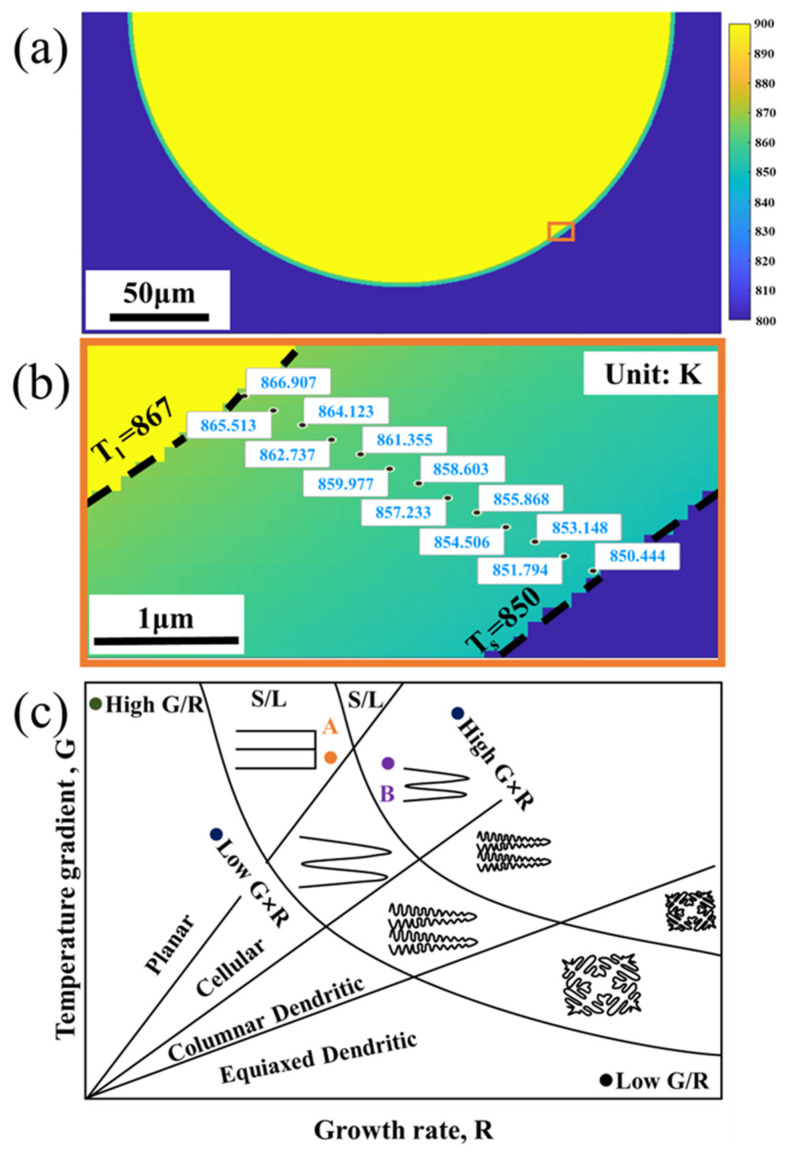
The influence of solidification conditions on the formed microstructure. (**a**) A modeled temperature field within a melt pool; (**b**) a close-up of the orange box in panel (**a**); (**c**) solidification microstructure selection map (adapted from [2]).

The LSZ of the LPBF AlSi10Mg sample corresponds to the liquid solidified zone of the melt pool, and the formation of the microstructure is correlated with the thermal gradient (*G*) and growth rate (*R*). Using the Rosenthal equation, it is possible to approximate the temperature field in the cross-section of melt pool, as well as *G* and *R* at the solidification front, as carried out in the reference [46]. The approximate temperature field of melt pool is described as follows:

(1) $$T=T_{0}+\frac{\alpha_{0}\cdot P}{2\pi \cdot \kappa \cdot \sqrt{x^{2}+y^{2}+z^{2}}}\cdot exp\left(-\frac{V}{2\alpha }\cdot \left(x+\sqrt{x^{2}+y^{2}+z^{2}}\right)\right)$$

in the equation, the *x*, *y*, and *z* directions represent the scan, transverse, and depth di-rection, respectively. *T_0_* is the ambient temperature (300 K), *α_0_* the absorbed power coefficient, *P* the laser power (150 W), *κ* the thermal conductivity, *V* the scan speed (400 mm/s), and *α* the thermal diffusivity. With reference to the work of Jiang et al. [46], *α_0_* is tentatively taken to be 0.625, *κ* to be 100 W/(m·K), and *α* to be 4 × 10^−5^ m^2^/s. Substituting these values into Equation (1), an approximated temperature field of the melt pool is attainable. For a convenient exhibition, a cross-section of the approximated temperature field (at *x* = 0) is displayed in Figure 10a. In order to more intuitively show the distribution of the three zones, temperatures greater than the liquidus temperature (T_l_~867 K) are set to 900 K, and temperatures lower than the solidus temperature (T_s_~850 K) are set to 800 K. In addition, the melt pool boundary is defined by the green zone, which is between the liquidus temperature and the solidus temperature. Figure 10b shows an enlarged view of the melt pool boundary of Figure 10a, in which the green zone is labeled with the modeled temperatures along the direction of heat transfer. *G* gradually increases from the melt pool center to the boundary and reaches the maximum value at the latter. Since the microstructure of the LPBF AlSi10Mg sample tends to grow in the direction of heat transfer, i.e., perpendicular to the melt pool boundary, *R* can thus be calculated. *R* decreases gradually from the center of the melt pool to the boundary and becomes almost zero at the melt pool boundary.

*G* and *R* effectively determine the morphology and size of the solidification structures. Many studies have proved that the *G/R* controls the constitutional supercooling which determines the solid/liquid interface stability and solidification morphology [47], with planar, cellular, columnar, and equiaxed dendritic structures appearing successively from high to low ratios. *G × R* (equivalent to the cooling rate) determines the characteristic length of the solidification microstructure, and generally a higher cooling rate leads to finer microstructural features [48]. The microstructural changes can be rationalized based on a metal solidification diagram (Figure 10c). At the edge of the melt pool, *R* is close to 0 and *G* is larger, corresponding to point ***A*** in Figure 10c, a region where compositions that are supercooled are difficult to form and grow in a planar-like structure. However, this zone exists only in a very small range at the edge of the melt pool and is easily overlooked. Pham et al. [49] reported a similar phenomenon in the high entropy alloys prepared via LPBF. Towards the interior of the melt pool, *R* increases and *G* decreases, and the solidification condition is likely to position at point ***B*** in Figure 10c, where fine cellular growth prevails. As a result of the variations in *G* and *R*, the microstructure of LSZ changes from a Si meshes structure formed by planar-like growth at the melt pool boundary to a fine cellular structure at the center. The cellular structure tends to elongate in the direction of growth, i.e., pointing from the melt pool boundary to the center. This also verifies that in Figure 4a, the network structure in LSZ1 perpendicular to the melt pool boundary and in LSZ2 perpendicular to plane of paper. The unstable cellular structure marked by the purple dashed line in Figure 4a is occasionally present at the LSZ boundary, which is strongly related to the shift in the growth direction shown in Figure 7c. As the growth direction changes, the cellular structure enters a transition zone of competing growth, resulting in chaotic growth of cellular structures in this zone. As the angle of change increases, the scale of unstable growth becomes longer.

Two explanations for the formation of MSZ at the melt pool boundary have been proposed in previous studies. One of them is that the supercooling in the melt pool changed due to the Gaussian distribution of the laser energy. The value of *G* reaches a maximum at the melt pool center and minimum at melt pool boundary. The value of *G × R* determines the characteristic length of the structure, which results in the formation of the MSZ at the melt boundary [48,50]. The change of *G × R* should be continuous throughout the melt pool, which leads to the transformation of the cellular structure which should be a gradual change from coarse to fine [22]. However, in the LPBF AlSi10Mg sample, the Si-rich network shows an abrupt change between the LAZ and MSZ. Another explanation is the increasing size of the cellular structure at overlaps due to remelting of the solidified material to form MSZ. But the thickness of the overlap zone between the two layers is much wider than the observed MSZ. In this paper, it is found that the formation of the MSZ microstructure of the LPBF AlSi10Mg sample is related to a semisolid state of the alloy. The temperature interval between the liquidus temperature (867 K) and the solidus temperature (850 K) may be used to define MSZ. Seen from Figure 10b, the distance between the simulated solidus and liquidus lines is ~2.5 μm, in good agreement with the width of MSZ observed in Figure 4c and Figure 7c. And it can be clearly found in Figure 4c that the cellular structure in MSZ has a similar orientation and comparable dimensions with respect to those of LSZ2. It is also easy to see from the statistical analysis in Figure 6b that the cellular structure in MSZ and the cellular structure in the matrix metal LSZ are close in the aspect ratio, despite a larger minor axis length for the former. The increase in size of the cellular structure is due to the influence of the new melt pool, where the matrix metal was reheated to a semisolid state and the cellular structure was coarsened [26]. Zoqui et al. [51] have also reported an analogous finding in their study of the A356 alloy; there was an increase in the size of the structure during the re-heating to the semisolid state.

The microstructure formation of the HAZ is correlated with the thermal influences on the solid alloy. The HAZ is located behind the MSZ, where the laser energy is insufficient to melt the matrix metal, but the heat needs to be dissipated from the matrix metal, and induces a heat treatment to the unmelted substrate near the melt pool boundary [26]. The current research only observe a narrow HAZ in the LPBF samples, which does not clearly show the evolution of the Si-rich network structure. To clarify the thermal effects more conveniently in the space, a laser remelting experiment was conducted to significantly extend the HAZ to show how the Si-rich network structure evolves in response to the dissipating heat, see Section 3.3. The Si-rich networks tend to have a fibrous shape, which is unstable due to a large specific interfacial area. When the structure was subjected to high temperatures, it broke and spheroidized, and the chemical potential gradient between the interfacial discontinuities and the adjacent area provided the energy required for solid-state atomic diffusion [52]. If exposed to a higher temperature or a longer dwell time, e.g., at a place in the HAZ right next to MSZ, the spheroidized particles became coarsened to further reduce the interfacial energy.

## 5. Conclusions

In this paper, the cellular structure, i.e., the Si-rich network structure of the LPBF AlSi10Mg sample has been investigated. We divide the whole structure into three zones: the liquid solidification zone (LSZ), the mushy solidification zone (MSZ), and the heat affected zone (HAZ). The boundary between LSZ and MSZ is identified by the change of grain growth direction and the Si-rich network advancing direction, and the boundary between MSZ and HAZ is identified by the start of the breakdown the of Si-rich network. Laser remelting experiments have clarified how the structure of the Si-rich evolves in the HAZ. The LSZ is subject to variations in solidification conditions, showing small planar growth at the boundary and large cellular growth in the center. The unstable growth in the cellular structure of the LSZ is induced when the growth orientation of neighboring melt pools is changed. The MSZ presents a mushy solidification that occurs to the Si-rich network of the matrix metal, and it shows a cellular structure larger than that of the base structure, while the shape and orientation is not changed. Tensile tests demonstrate that fractures propagate along the HAZ.

## Figures and Tables

**Table 1:** Chemical composition of the studied AlSi10Mg powders (wt. %).

Composition	Al	Si	Mg	Fe
AlSi10Mg	Bal.	9.91	0.36	0.10

## Data Availability

Data are contained within the article.

## References

[1] Lathabai S. (2018). Additive manufacturing of aluminium-based alloys and composites. Fundamentals of Aluminium Metallurgy.

[2] DebRoy T., Wei H.L., Zuback J.S., Mukherjee T., Elmer J.W., Milewski J.O., Beese A.M., Wilson-Heid A., De A., Zhang W. (2018). Additive manufacturing of metallic components—Process, structure and properties. Prog. Mater. Sci..

[3] Gu D.D., Meiners W., Wissenbach K., Poprawe R. (2013). Laser additive manufacturing of metallic components: Materials, processes and mechanisms. Int. Mater. Rev..

[4] Herzog D., Seyda V., Wycisk E., Emmelmann C. (2016). Additive manufacturing of metals. Acta Mater..

[5] Kotadia H.R., Gibbons G., Das A., Howes P.D. (2021). A review of Laser Powder Bed Fusion Additive Manufacturing of aluminium alloys: Microstructure and properties. Addit. Manuf..

[6] Martin J.H., Yahata B.D., Hundley J.M., Mayer J.A., Schaedler T.A., Pollock T.M. (2017). 3D printing of high-strength aluminium alloys. Nature.

[7] Kimura T., Nakamoto T., Ozaki T., Sugita K., Mizuno M., Araki H. (2019). Microstructural formation and characterization mechanisms of selective laser melted Al–Si–Mg alloys with increasing magnesium content. Mater. Sci. Eng. A.

[8] Sun Z., Tan X., Tor S.B., Yeong W.Y. (2016). Selective laser melting of stainless steel 316L with low porosity and high build rates. Mater. Des..

[9] Wang D., Song C., Yang Y., Bai Y. (2016). Investigation of crystal growth mechanism during selective laser melting and mechanical property characterization of 316L stainless steel parts. Mater. Des..

[10] Ma M., Wang Z., Zeng X. (2017). A comparison on metallurgical behaviors of 316L stainless steel by selective laser melting and laser cladding deposition. Mater. Sci. Eng. A.

[11] Cao S., Chen Z., Lim C.V.S., Yang K., Jia Q., Jarvis T., Tomus D., Wu X. (2017). Defect, microstructure, and mechanical property of Ti-6Al-4V alloy fabricated by high-power selective laser melting. JOM.

[12] Thijs L., Verhaeghe F., Craeghs T., Van Humbeeck J., Kruth J.P. (2010). A study of the microstructural evolution during selective laser melting of Ti–6Al–4V. Acta Mater..

[13] Geiger F., Kunze K., Etter T. (2016). Tailoring the texture of IN738LC processed by selective laser melting (SLM) by specific scanning strategies. Mater. Sci. Eng. A.

[14] Kunze K., Etter T., Grässlin J., Shklover V. (2015). Texture, anisotropy in microstructure and mechanical properties of IN738LC alloy processed by selective laser melting (SLM). Mater. Sci. Eng. A.

[15] Sun S.H., Hagihara K., Nakano T. (2018). Effect of scanning strategy on texture formation in Ni-25 at.% Mo alloys fabricated by selective laser melting. Mater. Des..

[16] Aversa A., Lorusso M., Cattano G., Manfredi D., Calignano F., Ambrosio E.P., Biamino S., Fino P., Lombardi M., Pavese M. (2017). A study of the microstructure and the mechanical properties of an Al-Si-Ni alloy produced via selective laser melting. J. Alloys Compd..

[17] Rao H., Giet S., Yang K., Wu X., Davies C.H. (2016). The influence of processing parameters on aluminium alloy A357 manufactured by Selective Laser Melting. Mater. Des..

[18] Wu J., Wang X.Q., Wang W., Attallah M.M., Loretto M.H. (2016). Microstructure and strength of selectively laser melted AlSi10Mg. Acta Mater..

[19] Li X.P., Ji G., Chen Z., Addad A., Wu Y., Wang H.W., Vleugels J., Van Humbeeck J., Kruth J.P. (2017). Selective laser melting of nano-TiB_2_ decorated AlSi10Mg alloy with high fracture strength and ductility. Acta Mater..

[20] Ai X., Wang J., Wen T., Yang F., Dong X., Yang H., Ji S. (2022). A high Fe-containing AlSi12 alloy fabricated by laser powder bed fusion. J. Mater. Res. Technol..

[21] Li X.P., Wang X.J., Saunders M., Suvorova A., Zhang L.C., Liu Y.J., Fang M.H., Huang Z.H., Sercombe T.B. (2015). A selective laser melting and solution heat treatment refined Al–12Si alloy with a controllable ultrafine eutectic microstructure and 25% tensile ductility. Acta Mater..

[22] Wei P., Wei Z., Chen Z., Du J., He Y., Li J., Zhou Y. (2017). The AlSi10Mg samples produced by selective laser melting: Single track, densification, microstructure and mechanical behavior. Appl. Surf. Sci..

[23] Liu Y.J., Liu Z., Jiang Y., Wang G.W., Yang Y., Zhang L.C. (2018). Gradient in microstructure and mechanical property of selective laser melted AlSi10Mg. J. Alloys Compd..

[24] Thijs L., Kempen K., Kruth J.P., Van Humbeeck J. (2013). Fine-structured aluminium products with controllable texture by selective laser melting of pre-alloyed AlSi10Mg powder. Acta Mater..

[25] Kim D.K., Hwang J.H., Kim E.Y., Heo Y.U., Woo W., Choi S.H. (2017). Evaluation of the stress-strain relationship of constituent phases in AlSi10Mg alloy produced by selective laser melting using crystal plasticity FEM. J. Alloys Compd..

[26] Liu X., Zhao C., Zhou X., Shen Z., Liu W. (2019). Microstructure of selective laser melted AlSi10Mg alloy. Mater. Des..

[27] Patakham U., Palasay A., Wila P., Tongsri R. (2021). MPB characteristics and Si morphologies on mechanical properties and fracture behavior of SLM AlSi10Mg. Mater. Sci. Eng. A.

[28] Dilip J.J.S., Zhang S., Teng C., Zeng K., Robinson C., Pal D., Stucker B. (2017). Influence of processing parameters on the evolution of melt pool, porosity, and microstructures in Ti-6Al-4V alloy parts fabricated by selective laser melting. Prog. Addit. Manuf..

[29] Yan Q., Song B., Shi Y. (2020). Comparative study of performance comparison of AlSi10Mg alloy prepared by selective laser melting and casting. J. Mater. Sci. Technol..

[30] Prashanth K.G., Eckert J. (2017). Formation of metastable cellular microstructures in selective laser melted alloys. J. Alloys Compd..

[31] Liu W., Ye L., Liu K. (2011). Micro-nano scale ripples on metallic glass induced by laser pulse. J. Appl. Phys..

[32] Li W., Li S., Liu J., Zhang A., Zhou Y., Wei Q., Yan C., Shi Y. (2016). Effect of heat treatment on AlSi10Mg alloy fabricated by selective laser melting: Microstructure evolution, mechanical properties and fracture mechanism. Mater. Sci. Eng. A.

[33] Gordon J.V., Narra S.P., Cunningham R.W., Liu H., Chen H., Suter R.M., Beuth J.L., Rollett A.D. (2020). Defect structure process maps for laser powder bed fusion additive manufacturing. Addit. Manuf..

[34] Qin H., Fallah V., Dong Q., Brochu M., Daymond M.R., Gallerneaultet M. (2018). Solidification pattern, microstructure and texture development in Laser Powder Bed Fusion (LPBF) of Al10SiMg alloy. Mater. Charact..

[35] Kempf A., Hilgenberg K. (2021). Influence of heat treatments on AlSi10Mg specimens manufactured with different laser powder bed fusion machines. Mater. Sci. Eng. A.

[36] Prashanth K.G., Scudino S., Klauss H.J., Surreddi K.B., Löber L., Wang Z., Chaubey A.K., Kühn U., Eckert K.H. (2014). Microstructure and mechanical properties of Al–12Si produced by selective laser melting: Effect of heat treatment. Mater. Sci. Eng. A.

[37] Alghamdi F., Song X., Hadadzadeh A., Shalchi-Amirkhiz B., Mohammadi M., Haghshenas M. (2020). Post heat treatment of additive manufactured AlSi10Mg: On silicon morphology, texture and small-scale properties. Mater. Sci. Eng. A.

[38] Zhou Y., Wen S., Wang C., Duan L., Wei Q., Shi Y. (2019). Effect of TiC content on the Al-15Si alloy processed by selective laser melting: Microstructure and mechanical properties. Opt. Laser Technol..

[39] Gao C., Liu Z., Xiao Z., Zhang W., Wong K., Akbarzadeh A.H. (2021). Effect of heat treatment on SLM-fabricated TiN/AlSi10Mg composites: Microstructural evolution and mechanical properties. J. Alloys Compd..

[40] Amir B., Grinberg E., Gale Y., Sadot O., Samuha S. (2021). Influences of platform heating and post-processing stress relief treatment on the mechanical properties and microstructure of selective-laser-melted AlSi10Mg alloys. Mater. Sci. Eng. A.

[41] Sélo R.R.J., Catchpole-Smith S., Maskery I., Ashcroft I., Tuck C. (2020). On the thermal conductivity of AlSi10Mg and lattice structures made by laser powder bed fusion. Addit. Manuf..

[42] Yang P., Deibler L.A., Bradley D.R., Stefan D.K., Carroll J.D. (2018). Microstructure evolution and thermal properties of an additively manufactured, solution treatable AlSi10Mg part. J. Mater. Res..

[43] Zhao L., Santos Macías J.G., Douillard T., Li Z., Simar A. (2021). Unveiling damage sites and fracture path in laser powder bed fusion AlSi10Mg: Comparison between horizontal and vertical loading directions. Mater. Sci. Eng. A.

[44] Kou S. (2003). Welding Metallurgy.

[45] Jiang Z., Xu P., Liang Y., Liang Y. (2023). Deformation effect of melt pool boundaries on the mechanical property anisotropy in the SLM AlSi10Mg. Mater. Today Adv..

[46] Jiang A., Zhang X., Xu Z., Long Y., Wang X. (2024). A model for solute evolution in laser powder bed fused Al-Si alloys and its application to improve thermal conductivity. Acta Mater..

[47] Hadadzadeh A., Amirkhiz B.S., Langelier B., Li J., Mohammadi M. (2021). Microstructural consistency in the additive manufactured metallic materials: A study on the laser powder bed fusion of AlSi10Mg. Addit. Manuf..

[48] Yadroitsev I., Krakhmalev P., Yadroitsava I., Johansson S., Smurov I. (2013). Energy input effect on morphology and microstructure of selective laser melting single track from metallic powder. J. Mater. Process. Technol..

[49] Pham M.S., Dovgyy B., Hooper P.A., Gourlay C.M., Piglione A. (2020). The role of side-branching in microstructure development in laser powder-bed fusion. Nat. Commun..

[50] Trevisan F., Calignano F., Lorusso M., Pakkanen J., Aversa A., Ambrosio E.P., Lombardi M., Fino P., Manfredi D. (2017). On the selective laser melting (SLM) of the AlSi10Mg alloy: Process, microstructure, and mechanical properties. Materials.

[51] Zoqui E., Shehata M., Paes M., Kao V., Es-Sadiqi E. (2002). Morphological evolution of SSM A356 during partial remelting. Mater. Sci. Eng. A.

[52] Alghamdi F., Haghshenas M. (2019). Microstructural and small-scale characterization of additive manufactured AlSi10Mg alloy. SN Appl. Sci..

